# Hybrid Intelligent Nonlinear Optimization for FDA-MIMO Passive Microwave Arrays Radar on Static Platforms

**DOI:** 10.3390/mi17010027

**Published:** 2025-12-25

**Authors:** Yimeng Zhang, Wenxing Li, Bin Yang, Chuanji Zhu, Kai Dong

**Affiliations:** 1 College of Information and Communication Engineering, Harbin Engineering University, Harbin 150001, China; 2012082228@hrbeu.edu.cn (Y.Z.); liwenxing@hrbeu.edu.cn (W.L.); 2 School of Cyberspace, Hangzhou Dianzi University, Hangzhou 310018, China; 3 School of Electronic and Information Engineering, Heilongjiang University of Science and Technology, Harbin 150022, China; zhuchuanjie@hrbeu.edu.cn; 4 Center for Wireless Communications, University of Oulu, 90570 Oulu, Finland; kai.dong@oulu.fi

**Keywords:** passive microwave arrays, FDA-MIMO, nonlinear frequency offsets, hybrid intelligent optimization, two-dimensional beam focusing, interference suppression

## Abstract

Microwave, millimeter-wave, and terahertz devices are fundamental to modern 5G/6G communications, automotive imaging radar, and sensing systems. As essential RF front-end elements, passive microwave array components on static platforms remain constrained by fixed geometry and single-frequency excitation, leading to limited spatial resolution and weak interference suppression. Phase-steered arrays offer angular control but lack range-dependent response, preventing true two-dimensional focusing. Frequency-Diverse Array Multiple-Input Multiple-Output (FDA-MIMO) architectures introduce element-wise frequency offsets to enrich spatial–spectral degrees of freedom, yet conventional linear or predetermined nonlinear offsets cause range–angle coupling, periodic lobes, and restricted beamforming flexibility. Existing optimization strategies also tend to target single objectives and insufficiently address target- or scene-induced perturbations. This work proposes a nonlinear frequency-offset design for passive microwave arrays using a Dingo–Gray Wolf hybrid intelligent optimizer. A multi-metric fitness function simultaneously enforces sidelobe suppression, null shaping, and frequency-offset smoothness. Simulations in static scenarios show that the method achieves high-resolution two-dimensional focusing, enhanced interference suppression, and stable performance under realistic spatial–spectral mismatches. The results demonstrate an effective approach for improving the controllability and robustness of passive microwave array components on static platforms.

## 1. Introduction

In modern wireless systems, microwave, millimeter-wave, and terahertz devices serve as core enabling technologies for 5G/6G communications, automotive imaging radar, biomedical sensing, and security monitoring. As critical components of radio-frequency (RF) front-ends, passive microwave elements significantly influence overall system performance. Although advanced fabrication techniques such as MEMS and 3D printing have greatly improved the manufacturing precision and integration capability of passive devices, passive microwave array components [1], deployed on static platforms (e.g., ground-based or tower-mounted installations) still face inherent limitations in spatial resolution and interference suppression due to their fixed geometry and single-frequency excitation. Conventional phase-steered arrays provide angular control but lack range-dependent response, preventing two-dimensional point focusing [2]. Consequently, passive arrays remain limited by their intrinsic electromagnetic degrees of freedom, often demonstrating constrained beamforming flexibility, elevated sidelobes, and high sensitivity to perturbations in realistic electromagnetic environments. These challenges highlight the need for component-level design methodologies that enhance controllability, spatial–spectral flexibility, and robustness beyond what fixed-geometry passive arrays can inherently provide.

Frequency-Diverse Array (FDA) [3] and Multiple-Input Multiple-Output (MIMO) [4] techniques offer complementary mechanisms for expanding controllable degrees of freedom in passive microwave array components. FDA introduces element-wise frequency offsets that extend the array response from a purely angular domain to a coupled range–angle domain, enabling additional spatial–spectral control at the component level. MIMO contributes virtual-aperture synthesis and waveform diversity, improving effective aperture and design flexibility. Moreover, by replacing large-scale phase-shifting networks with frequency-offset-based control, FDA-MIMO architectures inherently reduce front-end hardware complexity, providing a favorable basis for array miniaturization and integrated implementation, and establishing a multidimensional foundation for advanced passive array design.

In FDA and FDA-MIMO architectures, the frequency-offset (FO) design is the core mechanism that determines the spatial–spectral response and interference resilience of passive microwave array components. Traditional linear FO is easy to implement but inherently introduces range–angle coupling and periodic grating lobes, restricting the achievable degrees of freedom and limiting the two-dimensional control required in static-platform sensing. Various fixed-form nonlinear FOs (such as logarithmic [5], random [6], and Costas sequence-based designs [7]) have been developed to reduce coupling, and windowing strategies (e.g., Hamming window [8], Taylor window [9], and Chebyshev window [10]) have been applied to suppress periodic lobes. However, the former provide limited methodological flexibility, while the latter preserve the underlying linear structure, leaving fundamental coupling mechanisms unresolved at the array-design level. Subsequent extensions using symmetric or random perturbations to modify logarithmic FO.In [11,12] hybrid Taylor window–logarithmic function constructions [13], “window-shaped” design (sinc function (SIC) and weighted sinc function (WSC)) [14], or alternative structural substitutions (e.g., minimum-redundancy arrangements [15] or random power-increasing permutations (RPFM) [16]) offer scenario-specific improvements but remain constrained by pre-defined functional forms. Under static-platform conditions—characterized by fixed interference layouts and unavoidable array imperfections—these limitations hinder comprehensive optimization of focusing performance, sidelobe and grating-lobe behavior, and robustness, thereby motivating the need for more flexible and design-driven FO methodologies.

Intelligent optimization provides one way to overcome the intrinsic rigidity of fixed or semi-fixed FO structures by directly searching the FO space and enabling flexible nonlinear configurations. Prior works have applied genetic algorithms (GAs) to tune sinusoidal or logarithmic offsets [17,18], while particle swarm optimization (PSO) has been stated to have enhanced performance compared to GA [19], to design polynomial-based offsets [20], and to maximize maximum signal-to interference-plus-noise ratio (SINR) [21]. Although these approaches demonstrate performance gains, most remain confined to FDA architectures and employ a single optimization algorithm, thereby underutilizing the multidimensional controllable degrees of freedom inherent to FDA-MIMO components. Moreover, the associated fitness functions typically focus on a single objective (such as sidelobe reduction or mainlobe shaping) without providing a coordinated design strategy capable of jointly addressing focusing accuracy, interference suppression, and practical implementability.

To establish representative baselines for comparative evaluation, this study adopts four nonlinear FO strategies: the fixed-function SIC [14], the weighted fixed-function WSC [14], the custom-function RPFM [16], and the optimization-based PSO approach [19]. Together, these methods span the major categories of nonlinear FO design—from deterministic analytical formulations to intelligent optimization—and were originally proposed for conventional FDA configurations. Although developed under standard FDA architectures, their underlying FO formulations can be directly applied to FDA-MIMO arrays, enabling a fair and informative comparison.

Building upon these baselines and to address the limitations, this work develops a hybrid-intelligent nonlinear FO optimization framework (denoted as DGW), primarily constructed on the Dingoes Optimization Algorithm (DOA) [22]. The proposed DGW method optimizes nonlinear FOs for FDA-MIMO arrays operating on static remote-sensing platforms, enabling highs-resolution, interference-resilient, and spatial-spectrally flexible beamforming. The main contributions are as follows:

- DGW Optimization Framework
  – A Tent–Logistic–Cosine (TLC) hybrid chaotic mapping strategy [23] is incorporated into the initialization stage, enhancing population diversity and improving exploration stability under static-platform electromagnetic variability.
  – Within the Group Attack phase of DOA, the hierarchical leadership mechanism of GWO is integrated to preserve the global search capability while accelerating convergence. An additional elite-learning component further guides the population toward high-quality regions, improving overall solution optimality.
  – A multi-objective fitness function is formulated to jointly enforce mainlobe sharpness, maximum sidelobe suppression, multi-level sidelobe management, and FO smoothness. The maximum-sidelobe term is adaptively configured for interference-free versus interference-present conditions, enabling global sidelobe control and interference-direction nulling within a unified optimization model.
- The minimum variance distortionless response (MVDR) beamformer is employed to evaluate robustness under four mismatched conditions. Results show that the DGW-optimized FOs maintain significantly higher output SINR compared with SIC, WSC, RPFM, and PSO, demonstrating strong stability for passive array components on static platforms.

The remainder of this paper is organized as follows. Section 2 reviews the FDA-MIMO signal model, the output-SINR formulation, and the detailed design procedure of the proposed DGW-based FO optimization methodology. Section 3 presents the simulation results and performance analysis. Section 4 concludes the paper.

## 2. Materials and Methods

**Figure 1:**
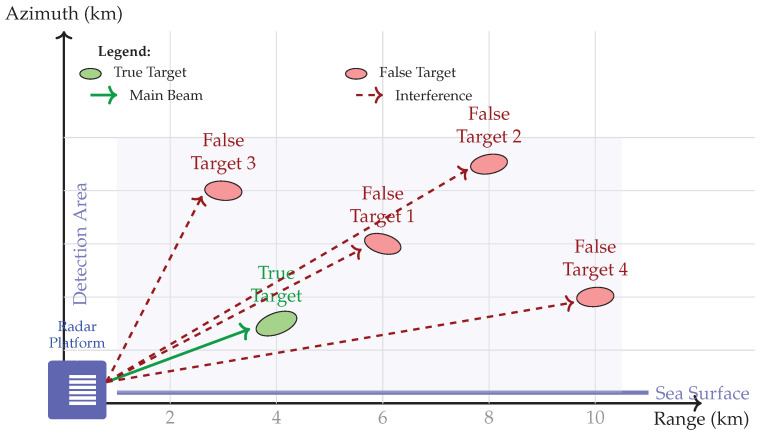
Shore-based static-platform FDA-MIMO radar configuration for maritime target detection.

In this study, the detection of sea-surface targets from a shore-based static remote-sensing platform is adopted as a representative application scenario to systematically evaluate FDA-MIMO radar performance. As illustrated in Figure 1, the platform is positioned along the coastline, providing a fixed and stable observational geometry. By optimizing the nonlinear FO configuration, the proposed method achieves enhanced beamforming performance, enabling high-resolution detection and discrimination of maritime targets. The scenario includes one real vessel target and four decoy targets acting as jamming sources. The real target represents the actual vessel of interest, while the decoys emulate either other vessels or potential electronic interference encountered under practical maritime conditions. All targets are modeled as point sources, consistent with standard radar signal processing assumptions and sufficient for evaluating the core system performance. The targets are deliberately distributed across the range–azimuth plane, with the real target located in a representative forward position and the decoys positioned at distinct ranges and azimuth angles. This spatial layout imposes stringent requirements on two-dimensional resolution and provides a representative environment for assessing the effectiveness of the proposed frequency-offset optimization.

### 2.1. FDA-MIMO Radar Signal Model

**Figure 2:**
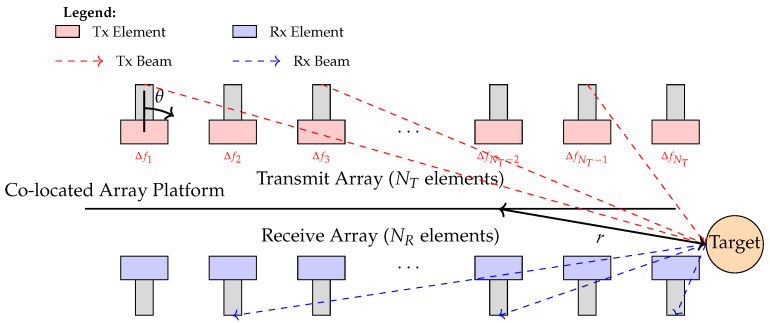
FDA-MIMO radar system signal model.

Figure 2 depicts the FDA-MIMO radar system, consisting of a transmitting array of $N_{T}$ elements and a receiving array of $N_{R}$ elements, both arranged as uniform linear arrays (ULAs) with the inter-element spacings of $d_{T}$ and $d_{R}$. The carrier frequency of the *l*-th transmitting element, $f_{l}$, is given by

(1) $$f_{l}=f_{c}+\Delta f_{l},l=1,2,\ldots ,N_{T},$$

where $f_{c}$ is the reference carrier frequency and $\Delta f_{l}=k_{l}\Delta f$ denotes the designed frequency offset for the *l*-th element that satisfies $\Delta f\ll f_{c}$. The proper selection of $k=\left[k_{1},k_{2},\ldots ,k_{N_{T}}\right]$ (FO coefficients) is crucial for achieving range–angle decoupling and improving the two-dimensional resolution performance of the FDA-MIMO radar system.

The transmit signal of the *l*-th transmitting element is shown as

(2) $$S_{l}\left(t\right)=s_{l}\left(t\right)e^{j2\pi f_{l}t},$$

where $s_{l}\left(t\right)$ denotes the complex envelope of the *l*-th transmit element, following $\int s_{l}\left(t\right)s_{n}^{*}\left(t\right)\;dt=1$.

Under the far-field assumption, a scattering point located at (θ,r), where θ is the azimuth angle measured relative to the radar’s boresight direction, and *r* denotes the propagation distance from the radar platform to the scattering point. The time delay of the signal transmitted by the *l*-th transmitting element and received by the *n*-th receiving element after scattering from the target is given by

(3) $$\tau_{l,n}=\frac{2r}{c}-\left(l-1\right)d_{T}\frac{\sin\theta }{c}-\left(n-1\right)d_{R}\frac{\sin\theta }{c},$$

where *c* is the speed of light.

Then, the backscattered signal from this scattering point can be expressed as

(4) $$S_{l,n}\left(t\right)=\sum_{l=1}^{N_{T}}\alpha \;S_{l}\left(t-\tau_{l,n}\right)e^{j2\pi f_{l}\left(t-\tau_{l,n}\right)},$$

where α is the signal attenuation incurred during propagation.

By analyzing the phase term associated with the target echo after matched-filter processing, the transmit and receive steering vectors (SVs) of the FDA-MIMO radar can be constructed. These SVs characterize the range–angle–frequency coupling behavior induced by the FOs. The transmit SV $a_{T}\left(\theta ,r\right)$ and receive SV $a_{R}\left(\theta \right)$ associated with the signal, which are defined as follows:

(5) $$\begin{array}{cc} a_{T}\left(\theta ,r\right)= & a_{T_{\theta }}\left(\theta \right)⊙a_{T_{r}}\left(r\right)\\ = & {\left[1,e^{j2\pi \frac{d_{T}}{\lambda }\sin\theta },\ldots ,e^{j2\pi \left(N_{T}-1\right)\frac{d_{T}}{\lambda }\sin\theta }\right]}^{T}\\ \; & ⊙{\left[e^{-j2\pi \Delta f_{1}\frac{2r}{c}},e^{-j2\pi \Delta f_{2}\frac{2r}{c}},\ldots ,e^{-j2\pi \Delta f_{N_{T}}\frac{2r}{c}}\right]}^{T}, \end{array}$$

where ⊙ denotes the Hadamard product, $a_{T_{r}}\left(r\right)$ is the transmit range SV, $a_{T_{\theta }}\left(\theta \right)$ is the transmit angle SV, and $\lambda =c/f_{c}$ denotes the carrier wavelength:

(6) $$a_{R}\left(\theta \right)={\left[1,e^{j2\pi \frac{d_{R}}{\lambda }}\sin\theta ,\ldots ,e^{j2\pi \left(N_{R}-1\right)\frac{d_{R}}{\lambda }}\sin\theta \right]}^{T}.$$

Therefore, the SV of FDA-MIMO radar is shown as

(7) $$a\left(\theta ,r\right)=a_{T}\left(\theta ,r\right)⊗a_{R}\left(\theta \right),$$

where ⊗ denotes the Kronecker product.

The received signal *S*, as a set of $S_{l,n}$, is shown below:

(8) $$\begin{array}{cc} S & ={\left[S_{1,1},S_{1,2},\ldots ,S_{1,N_{R}},S_{2,1},\ldots ,S_{l,n}\ldots ,S_{N_{T},N_{R}}\right]}^{T}\\ \; & =\xi \;e^{-j2\pi f_{c}\frac{2r}{c}}a_{T}\left(\theta ,r\right)⊗a_{R}\left(\theta \right). \end{array}$$

where ξ is the target scattering coefficient.

Then, the two-dimensional array factor of the FDA-MIMO radar is given by

(9) $$AF\left(\theta ,r\right)=w^{H}a\left(\theta ,r\right),$$

where w denotes the complex weighting coefficient, which ensures that the beampattern achieves its maximum at the desired signal location $\left(\theta_{0},R_{0}\right)$.

Accordingly, the normalized transmit–receive beampattern gain of the FDA-MIMO system can be expressed as

(10) $$G\left(\theta ,r\right)={\left|AF(\theta ,r)\right|}^{2}.$$

### 2.2. Hybrid Intelligent Optimization for FDA-MIMO Beamforming

To realize two-dimensional point-focused beamforming in FDA-MIMO arrays, this study adopts a nonlinear frequency-offset design to overcome the inherent limitations of linear offsets, including range–angle coupling and periodic grating lobes. The design problem is formulated as a hybrid intelligent optimization task for FO coefficient selection. The section is structured into two parts: the integration of optimization algorithms and the formulation of the multi-objective fitness function.

#### 2.2.1. DGW Optimizaiton Algorithm

As the core component of the DGW hybrid optimization framework, the Dingoes Optimization Algorithm (DOA) [22] exhibits strong capability for handling multi-parameter optimization tasks. The positions of *M* dingoes in the population initialization stage are defined as follows:

(11) $$\begin{array}{c} \mathrm{Pos}={\left[{\mathrm{Pos}}_{1},\ldots ,{\mathrm{Pos}}_{m},\ldots ,{\mathrm{Pos}}_{M}\right]}^{T}, \end{array}$$

where

(12) $${\mathrm{Pos}}_{m}=\left[pos_{m}^{1},pos_{m}^{2},\ldots ,pos_{m}^{N_{T}}\right],m=1,2,\ldots ,M.$$

Conventional random initialization often leads to uneven population distribution and inadequate coverage of the solution space. To address this, the proposed method incorporates the TLC chaotic mapping strategy to enhance initialization quality. The corresponding formulation is given below:

(13) $$z\left(m+1\right)\;\;=\;\;\left\{\begin{array}{cc} \cos\;\left(\pi \left(2uz(m)+4(1-u)z(m)(1-z(m))-0.5\right)\right), & \;\;\;z(m)\;\;<\;0.5,\\ \cos\;\left(\pi \left(2u(1-z(m))+4(1-u)z(m)(1-z(m))-0.5\right)\right), & \;\;\;\mathrm{else}, \end{array}\right.\;\;\;\;u\;\;\in \;\;\left[0,1\right].$$

By combining three mappings through a weighted scheme, the proposed strategy retains their individual advantages while achieving a balanced and robust initialization mechanism [23].

Furthermore, each $pos_{m}^{l}$ in (12) for the *m*-th dingo has four behavioral mechanisms: Group Attack, Persecution, Scavenging, and Survival Rates. For each position update, one of the four behavioral strategies is selected and executed. These behaviors are introduced in detail as follows.

During the Group Attack behavior, the global information collaboration mechanism of DOA is efficiently integrated with the hierarchical leadership guidance of GWO, enhancing the balance between global exploration and local exploitation.

The DOA Group Attack component is shown as

(14) $$pos_{new_{doa},m}^{l}=q_{m}^{l}\beta_{1}-pos_{best}^{l},$$

where $\beta_{1}$ represents a uniformly distributed random variable within [−2,2], $pos_{best}^{l}$ represents the best search dingo found from the previous iteration, and $q_{m}^{l}$ is the distance between *b* dingoes and the current dingo, given by

(15) $$q_{m}^{l}=\frac{1}{b}\sum_{i\neq m}^{M}(pos_{i}^{l}-pos_{m}^{l}),$$

where *b* is the number of dingoes joining Group Attack which is a random integer between [2,M/2].

The GWO Leadership Guidance Components is represented as

(16) $$pos_{new_{gwo},m}^{l}=\frac{pos_{gwo_{1},m}^{l}+pos_{gwo_{2},m}^{l}+pos_{gwo_{3},m}^{l}}{3}.$$

Then, $pos_{gwo_{1},m}^{l},pos_{gwo_{2},m}^{l},pos_{gwo_{3},m}^{l}$ are shown as

(17) $$\begin{array}{cc} pos_{gwo_{1},m}^{l} & =pos_{best_{1}}^{l}-A_{1}\cdot \left|C_{1}\cdot pos_{best_{1}}^{l}-pos_{m}^{l}\right|,\\ pos_{gwo_{2},m}^{l} & =pos_{best_{2}}^{l}-A_{2}\cdot \left|C_{2}\cdot pos_{best_{2}}^{l}-pos_{m}^{l}\right|,\\ pos_{gwo_{3},m}^{l} & =pos_{best_{3}}^{l}-A_{3}\cdot \left|C_{3}\cdot pos_{best_{3}}^{l}-pos_{m}^{l}\right|, \end{array}$$

where $A_{1},A_{2},A_{3}$ represent a uniformly distributed random variable within $\left[-a_{1},a_{1}\right]$, $a_{1}$ is a constant initially set to 2, and decreases linearly from 2 to 0 as the algorithm iterates. $C_{1},C_{2},C_{3}$ represent a uniformly distributed random variable within [0,2], and $pos_{best_{1}}^{l}$, $pos_{best_{2}}^{l}$, and $pos_{best_{3}}^{l}$ represent the top three best search dingoes found from the previous iteration.

The Group Attack behavior is finally represented as

(18) $$pos_{new,m}^{l}=\left(1-\beta_{2}\right)\cdot pos_{new_{doa},m}^{l}+\beta_{2}\cdot pos_{new_{gwo},m}^{l}+\beta_{lrn}\cdot \left(pos_{best}^{l}-pos_{m}^{l}\right).$$

where $\beta_{2}$ denotes adaptive hybrid weighting, and $\beta_{lrn}$ is towards the optimal learning factor.

The Persecution behavior is expressed as

(19) $$pos_{new,m}^{l}=pos_{best}^{l}+\beta_{1}e^{\beta_{3}}\left(pos_{r}^{l}-pos_{m}^{l}\right)+\alpha_{1}⊗\mathrm{levy}\left(pos_{m}^{l}\right),$$

where $pos_{r}^{l}$ is a random dingo selected from the dingoes group, $\beta_{3}$ represents a uniformly distributed random variable within [−1,1], $\alpha_{1}\in U\left(0,1\right)$, and levy(·) denotes the Levy’s flight.

The Scavenger behavior is given by

(20) $$pos_{new,m}^{l}=0.5\left(e^{\beta_{3}}pos_{r}^{l}-{\left(-1\right)}^{s}pos_{m}^{l}\right),$$

where *s* is a random binary number.

The Dingoes’ Survival Rates behavior is defined as:

(21) $$\begin{array}{cc} pos_{new,m}^{l}= & pos_{best}^{l}+\left(pos_{r1}^{l}-{\left(-1\right)}^{s}pos_{r2}^{l}\right)/2\\ \; & +\alpha_{1}⊗\mathrm{levy}\left(pos_{m}^{l}\right), \end{array}$$

where $pos_{r1}^{l}$ and $pos_{r2}^{l}$ are two different random dingoes selected from the dingoes group.

#### 2.2.2. Design of the Fitness Function

To achieve two-dimensional point-focused beamforming and suppress sidelobes in undesired directions, the fitness function is formulated as a multi-objective optimization model that simultaneously considers sidelobe suppression, mainlobe enhancement, and the smoothness of the frequency-offset design.

This function is formulated as a weighted combination of four interrelated technical indicators, shown below:

(22) $$Fit\left(k\right)=w_{1}\cdot Fit_{\mathrm{WSLL}}\left(k\right)+w_{2}\cdot Fit_{\mathrm{MSLL}}\left(k\right)+w_{3}\cdot Fit_{\mathrm{MP}}\left(k\right)+w_{4}\cdot Fit_{\mathrm{TV}}\left(k\right),$$

where the *I* weighting coefficients of this subsection satisfy $\sum_{i=1}^{I}w_{i}=1$, and $w_{i}>0$.

For the Weighted Sidelobe Level (WSLL) metric, distinct weighting strategies are employed to effectively account for scenarios with and without interference. The combined WSLL formulation is expressed as follows:

(23) $$Fit_{\mathrm{WSLL}}\left(k\right)=\max_{\left(\theta ,r\right)\in \Omega_{\mathrm{SL}}}\left\{W(\theta ,r)\cdot G(\theta ,r)\right\}.$$

The mainlobe region $\Omega_{\mathrm{ML}}$ is given by

(24) $$\Omega_{\mathrm{ML}}=\left\{\left(\theta ,r\right)||\theta -\theta_{0}|\leq \Delta \theta_{\mathrm{ML}}\;\wedge \;\left|r-R_{0}\right|\leq \Delta r_{\mathrm{ML}}\right\},$$

where $\Delta \theta_{\mathrm{ML}}$ and $\Delta r_{\mathrm{ML}}$ represent the extent of the mainlobe’s expansion in the angular and distance dimensions, respectively. $\Omega_{\mathrm{SL}}$ is sidelobe level domain excluding the mainlobe region.

W(θ,r) is a direction-weighted function, considering the location and power of *J* interference sources:

(25) $$W\left(\theta ,r\right)=1+\sum_{j=1}^{J}\frac{G_{I,j}}{\max(G_{I})}\cdot \exp\left(-\frac{{\left(\theta -\theta_{j}\right)}^{2}}{2\sigma_{\theta }^{2}}-\frac{{\left(r-r_{j}\right)}^{2}}{2\sigma_{r}^{2}}\right),$$

where $\left(\theta_{j},r_{j}\right)$ and $G_{I,j}$ are the angle, distance and power of the *j*-th interference source, respectively, and $\sigma_{\theta }$ and $\sigma_{r}$ control the angle and range of tolerance.

In the absence of interference signals, the previous weighting strategy based on known interference locations is no longer applicable. To address this limitation, an intelligent spatial-threat-based weighting strategy is proposed in (26), shifting the design from targeted suppression to global intelligent suppression:

(26) $$W\left(\theta ,r\right)=W_{\mathrm{base}}+W_{\mathrm{range}}\left(\theta ,r\right)+W_{\mathrm{angle}}\left(\theta ,r\right)+W_{\mathrm{continuity}}\left(\theta ,r\right).$$

The four weights mentioned above represent the base weight, distance threat weight, angular threat weight, and spatial continuity weight, respectively:

(27) $$\begin{array}{cc} W_{\mathrm{base}} & =1,\\ W_{\mathrm{range}}\left(\theta ,r\right) & =\alpha_{r}\cdot \exp\left(-\frac{r-r_{\min}}{\beta_{r}\cdot \left(r_{\max}-r_{\min}\right)}\right),\\ W_{\mathrm{angle}}\left(\theta ,r\right) & =\alpha_{a}\cdot \exp\left(-\frac{\theta^{2}}{2\sigma_{a}^{2}}\right),\\ W_{\mathrm{continuity}}\left(\theta ,r\right) & =\alpha_{c}\cdot \frac{1}{1+|\nabla_{\theta }1|+|\nabla_{r}1|}, \end{array}$$

where $\alpha_{r}=2.0$, $\beta_{r}=0.2$, $r_{\min}$, and $r_{\max}$ are the minimum and maximum distances from the grid, respectively. $\alpha_{a}=1.5$, $\sigma_{a}=30^{\circ }$, and $\alpha_{c}=0.8$. $\nabla_{\theta }$ and $\nabla_{r}$ represent the gradient operators in the angular and distance directions, respectively.

The final weights after boundary correction are given by

(28) $$W_{\mathrm{final}}\left(\theta ,r\right)=W\left(\theta ,r\right)\cdot \left[1+\gamma \cdot \left(1-\frac{{\left|\theta \right|}^{2}}{\theta_{\max}^{2}}\right)\right],$$

where γ=0.3, $\theta_{\max}$ is the maximum angle.

The final weight is normalized to its maximum values:

(29) $$W\left(\theta ,r\right)=\frac{W_{\mathrm{final}}\left(\theta ,r\right)}{\max_{\theta ,r}W_{\mathrm{final}}\left(\theta ,r\right)}.$$

To prevent scenarios in which suppression of a single high-level sidelobe results in the elevation of others, a multi-level sidelobe suppression (MSLL) metric is introduced:

(30) $$Fit_{\mathrm{MSLL}}\left(k\right)=w_{1}\cdot \mathrm{MaxSLL}+w_{2}\cdot {\mathrm{Top}5}_{\mathrm{avg}}+w_{3}\cdot {\mathrm{Var}}_{\mathrm{SL}}+w_{4}\cdot \mathrm{PSLR},$$

where $\mathrm{MaxSLL}=\max_{\left(\theta ,r\right)\in \Omega_{\mathrm{SL}}}G\left(\theta ,r\right)$ is the maximum sidelobe level, ${\mathrm{Top}5}_{\mathrm{avg}}$ is average level of the top 5 sidelobes. ${\mathrm{Var}}_{\mathrm{SL}}$ represents the variance of the sidelobe level, reflecting the uniformity of the sidelobe distribution. PSLR denotes the peak sidelobe ratio.

To ensure that sidelobe suppression is not achieved at the expense of mainlobe quality, the Mainlobe Performance (MP) metric is expressed as

(31) $$Fit_{\mathrm{MP}}\left(k\right)=w_{1}\cdot \frac{1}{G_{\mathrm{peak}}}+w_{2}\cdot BW+w_{3}\cdot \left(1-S_{\mathrm{sym}}\right),$$

where $G_{\mathrm{peak}}$ is the peak power of the mainlobe, BW denotes the composite beamwidth, and $S_{\mathrm{sym}}$ is the mainlobe symmetry measurement.

(32) $$\begin{array}{cc} G_{\mathrm{peak}} & =\max_{\left(\theta ,r\right)\in \Omega_{\mathrm{ML}}}G\left(\theta ,r\right),\\ BW & =w_{1}\cdot BW_{\theta }+w_{2}\cdot BW_{r},\\ S_{\mathrm{sym}} & =1-\frac{∥G_{L}-G_{R}{∥}_{2}}{∥G_{L}{∥}_{2}+{∥G_{R}∥}_{2}}, \end{array}$$

where $BW_{\theta }$ and $BW_{r}$ represent the −3 dB beamwidths in the angular and range dimensions, respectively. The vectors $G_{L},G_{R}$ correspond to the power distributions on the left and right sides of the mainlobe.

In FDA-MIMO FO optimization, a total variation (TV) constraint is incorporated to ensure that the resulting frequency-offset configuration remains sufficiently smooth and practical for engineering implementation. The TV constraint is defined as the normalized sum of the absolute differences between consecutive FO coefficients:

(33) $$Fit_{\mathrm{TV}}\left(k\right)=\lambda_{1}\cdot \sum_{l=1}^{N_{T}-1}\left|\left(k_{l+1}-k_{l}\right)\cdot \Delta f\right|,$$

where $\lambda_{1}$ is the total variation penalty weighting coefficient.

Therefore, the complete optimization problem can be expressed as

(34) $$\begin{array}{cc} \min_{k}\; & Fit(k)\\ s.t.\; & k_{\min}\leq k_{l}\leq k_{\max},\;l=1,\ldots ,N_{T}\\ \; & k\in R^{N_{T}\times 1}. \end{array}$$

### 2.3. Output SINR

The MVDR beamformer is employed to assess the interference suppression capability and robustness of the proposed algorithm.

Consider J+1 uncorrelated narrowband far-field signals, consisting of one desired signal $S_{s}$ and *J* jamming sources $S_{J}$. Under this assumption, the complex received sample vector of the array at the g-th snapshot can be expressed as follows:

(35) $$\begin{array}{cc} S & =S_{s}+S_{J}+n\\ \; & =\xi \;e^{-j2\pi f_{c}\frac{2R_{0}}{c}}a_{T}\left(\theta_{0},R_{0}\right)⊗a_{R}\left(\theta_{0}\right)+\sum_{j=1}^{J}\xi \;e^{-j2\pi f_{c}\frac{2r_{j}}{c}}a_{T}\left(\theta_{j},r_{j}\right)⊗a_{R}\left(\theta_{j}\right)+n. \end{array}$$

where n denotes additive white Gaussian noise (AWGN) with zero mean and variance $\sigma_{n}^{2}I_{N_{T}N_{R}}$.

Following the beamforming operation, the output of the array beamformer can be expressed as

(36) $$X=w^{H}S.$$

The output SINR is given by

(37) $${\mathrm{SIN}R}_{\mathrm{out}}=\frac{\sigma_{s}^{2}{\left|w^{H}a\left(\theta_{0},R_{0}\right)\right|}^{2}}{w^{H}Rw},$$

where $\sigma_{s}^{2}$ is the signal power.

MVDR beamformers obtain the optimal weight vector by solving the following constraint optimization problem:

(38) $$\begin{array}{cccc} \; & \underset{w}{\mathrm{minimize}}\; & \; & E\left[{\left|X\right|}^{2}\right]=w^{H}Rw\\ \; & s.t.\; & \; & w^{H}a\left(\theta_{0},R_{0}\right)=1 \end{array}$$

where $w\in C^{N_{T}N_{R}\times 1}$ is the beamforming weight vector, $R\in C^{N_{T}N_{R}\times N_{T}N_{R}}$ denotes the covariance matrix of the jammings and noise signal, and $a\left(\theta_{0},R_{0}\right)\in C^{N_{T}N_{R}\times 1}$ is the SV of the target signal.

Applying the Lagrange multiplier method, the optimal weight vector for the MVDR beamformer can be obtained as follows:

(39) $$w_{\mathrm{MVDR}}=\frac{R^{-1}a\left(\theta_{0},R_{0}\right)}{a{\left(\theta_{0},R_{0}\right)}^{H}R^{-1}a\left(\theta_{0},R_{0}\right)}.$$

## 3. Results

To evaluate the proposed frequency-offset optimization method, an FDA-MIMO radar system operating on a static remote-sensing platform is considered in the simulations. The system employs a uniform linear array (ULA) with 15 transmit and 15 receive elements, spaced at half-wavelength intervals, $d_{R}=d_{T}=0.075$ m. $f_{c}=1$ GHz, and Δf=20 kHz. Assume the true target is located at $\left(100\;\mathrm{km},30^{\circ }\right)$. Four false targets are located at $\left(107\;\mathrm{km},30^{\circ }\right)$, $\left(102\;\mathrm{km},30^{\circ }\right)$, $\left(100\;\mathrm{km},36^{\circ }\right)$ and $\left(94\;\mathrm{km},20^{\circ }\right)$, which jointly span all typical configurations (covering same-angle/different-range, same-range/different-angle, and fully distinct range–angle positions). Some parameters of the proposed DGW algorithm are set as M=50,P=0.5,Q=0.7, and with a maximum number of 100 iterations (referred to [22]). In this section, the effectiveness and advantages of the proposed algorithm are validated through a comparative analysis against the SIC [14], RPFM [16], WSC [14], and PSO [19].

### 3.1. Range-Resolution Evaluation Under Ideal Conditions

**Figure 3:**
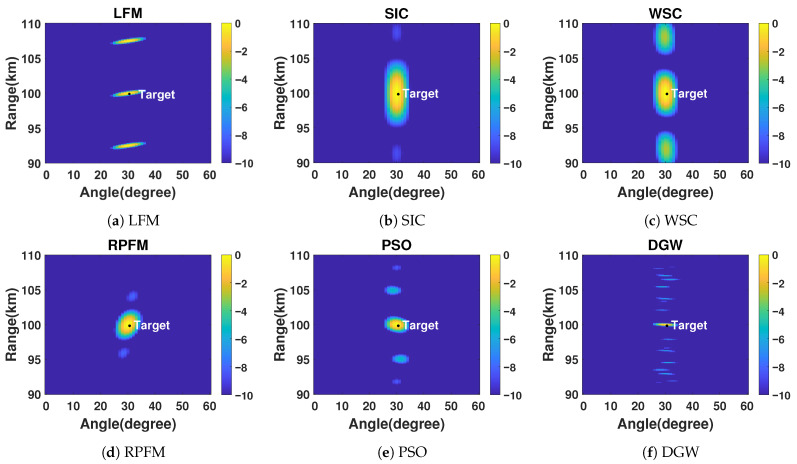
Transmit–receive beampatterns without any jamming.

Figure 3 shows the transmit–receive normalized beampatterns of six algorithms in the absence of interference. Compared with the range–angle coupling observed in Figure 3a under the traditional linear FO design, the other five algorithms effectively achieve decoupling and de-periodization, resulting in a more concentrated energy distribution. In particular, the proposed method (Figure 3f) provides the most pronounced energy focusing around the target location among all benchmarking schemes (Figure 3b–e), leading to a substantially narrower mainlobe in the range dimension. This improved focusing characteristic reflects the method’s enhanced range-resolution performance under ideal static remote-sensing conditions.

**Figure 4:**
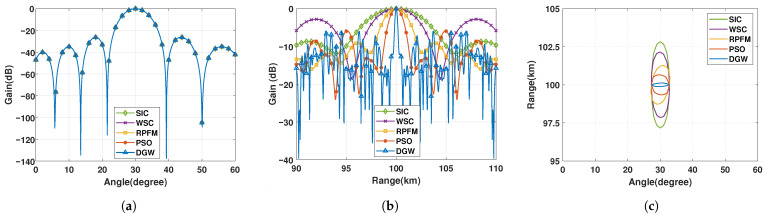
(**a**) Angle-dimensional beampattern at distance $R_{0}=100$ km. (**b**) Range-dimensional beampattern at angle $\theta_{0}=30^{\circ }$. (**c**) Half-power beampattern in range and angle dimensions.

The performance comparison is presented in a clear manner in Figure 4. Although all five algorithms produce almost the same beampatterns in the angular dimension, as illustrated in Figure 4a, a pronounced difference emerges in the range dimension, where the superior energy-focusing capability of the DGW algorithm can be clearly observed in Figure 4b. Figure 4c and Table 1 present the mainlobe width at the half-power beam level from graphical and numerical perspectives, respectively, thereby providing a concrete demonstration of the differences in energy-focusing capability among the algorithms. Specifically, in the range dimension, the WSC method reduces the mainlobe width by about 1.3 km relative to the SIC algorithm, while the RPFM method further narrows it by approximately 1.8 times. The proposed DGW algorithm, however, exhibits the most concentrated beam, with a mainlobe width 5 times narrower than the PSO algorithm (around 1.04 km) and nearly 10 times narrower than the RPFM algorithm (around 2.14 km), thereby demonstrating substantially enhanced range-direction focusing precision and offering improved high-resolution discrimination capability for static-platform passive microwave sensing.

### 3.2. Interference-Rejection Performance Under Ideal Conditions

**Figure 5:**
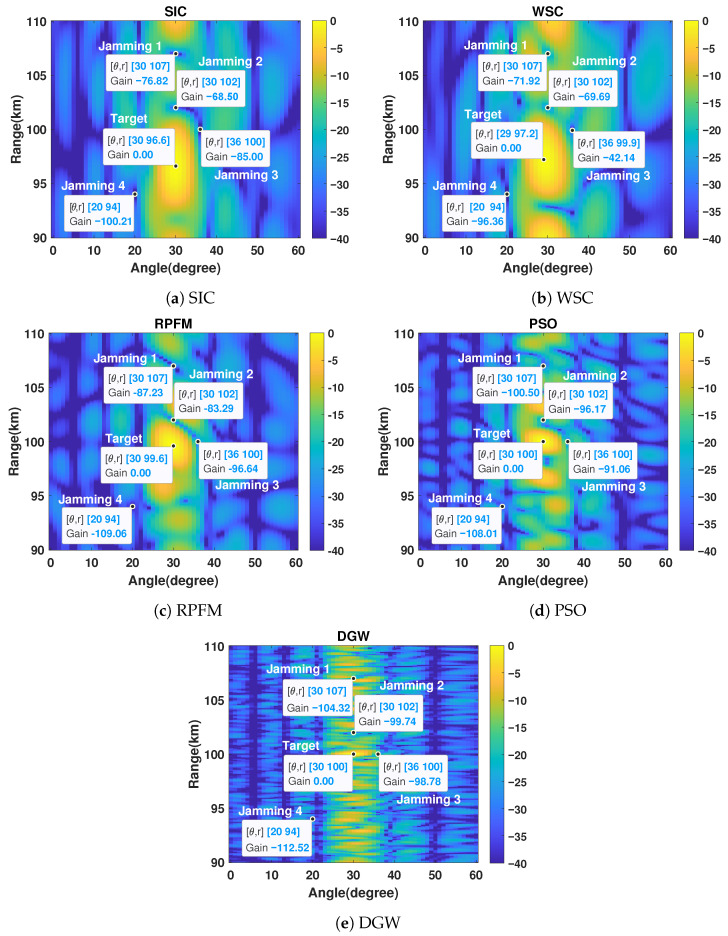
Transmit–receive beampatterns with four jammings.

To further evaluate the interference-suppression capability of each algorithm, a scenario with four jamming sources (false targets) is considered. As shown in Figure 5, all algorithms are able to suppress the interference at the four distinct jamming locations, thereby ensuring baseline detection reliability for static remote-sensing platforms operating under complex electromagnetic conditions. However, the proposed DGW algorithm (Figure 5e) not only achieves precise mainlobe-center alignment in both the range and angle dimensions but also maintains a noticeably narrower mainlobe width, resulting in more concentrated energy around the true target. The PSO algorithm (Figure 5d) attains comparable center alignment, yet its broader mainlobe reveals less effective focusing. In contrast, SIC and RPFM exhibit evident range-dimension shifts in the mainlobe center despite accurate angular alignment (Figure 5a,c), whereas the WSC method displays misalignment in both dimensions (Figure 5b). These results clearly show that DGW delivers the most accurate spatial–spectral control and interference-robust focusing among all candidates, effectively addressing the central challenge of static passive microwave arrays, where performance depends entirely on component-level frequency-offset design.

**Figure 6:**
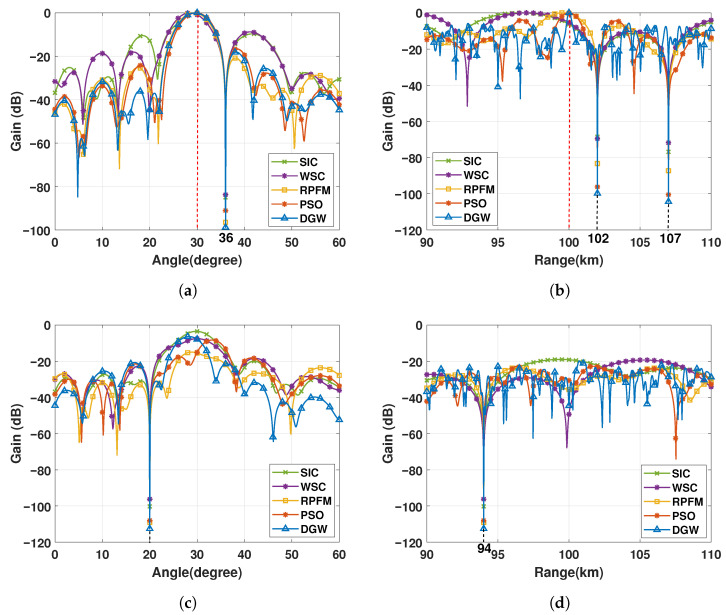
(**a**) Angle-dimensional beampattern at distance $R_{0}=100$ km. (**b**) Range-dimensional beampattern at angle $\theta_{0}=30^{\circ }$. (**c**) Angle-dimensional beampattern at distance r=94 km. (**d**) Range-dimensional beampattern at angle $\theta =20^{\circ }$.

Figure 6a shows that the mainlobe positions of the compared algorithms in the angular dimension are precise, except for the WSC algorithm, which exhibits a noticeable angular shift. All algorithms display generally similar mainlobe widths. In the range dimension illustrated in Figure 6b, the SIC, WSC, and RPFM methods exhibit pronounced mainlobe deviations, with SIC and WSC further showing dispersed energy distributions. In contrast, both PSO and DGW achieve accurate mainlobe localization, with the DGW algorithm producing a substantially narrower mainlobe, demonstrating superior energy-focusing capability. The emergent mainlobes of SIC and WSC in Figure 6c result from their excessively wide range-domain mainlobes combined with the aforementioned shifts. These results demonstrate that DGW achieves the most accurate mainlobe alignment and strongest energy concentration under interference, effectively leveraging component-level frequency-offset design to enhance spatial–spectral control of static-platform passive microwave arrays.

By jointly examining the numerical characteristics in Figure 5 and the beampatterns in Figure 6, it is evident that the proposed DGW algorithm consistently produces the deepest nulls against interference appearing at arbitrary locations. This demonstrates its superior interference-suppression capability compared with the other methods. PSO and RPFM exhibit moderate performance, whereas WSC and SIC achieve only shallow null depths and thus show the weakest suppression ability. These results collectively indicate that DGW delivers the strongest interference suppression and the most precise spatial–spectral control among the considered methods, effectively enhancing the performance of component-level passive microwave arrays on static platforms.

### 3.3. Robust Spatial–Spectral Performance Under Target- and Scene-Induced Perturbations

**Figure 7:**
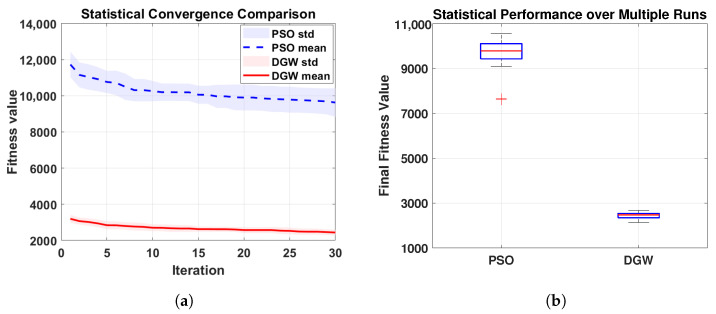
Convergence robustness and statistical stability comparison between DGW and PSO optimization algorithms. (**a**) Mean convergence curve with standard deviation under multiple independent runs. (**b**) Boxplot of final fitness values over multiple independent runs.

**Figure 8:**
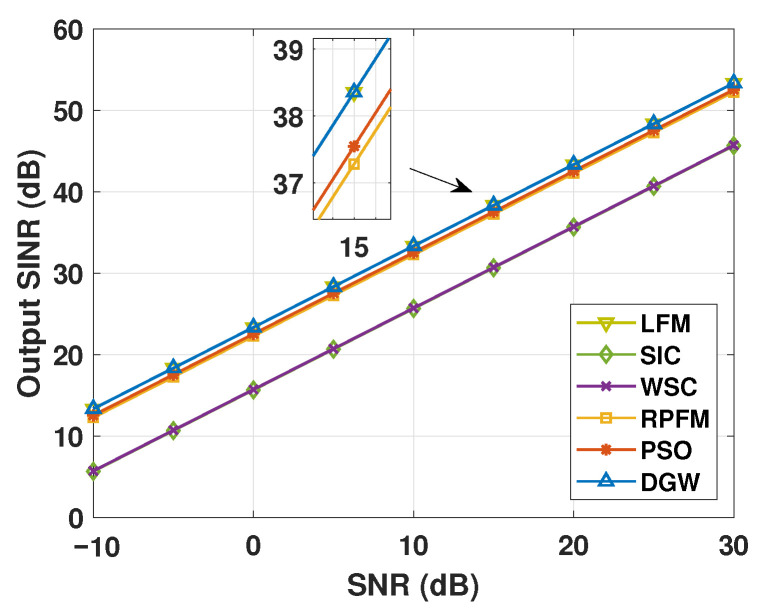
Comparison of output–input SNR for different algorithms under ideal conditions.

**Figure 9:**
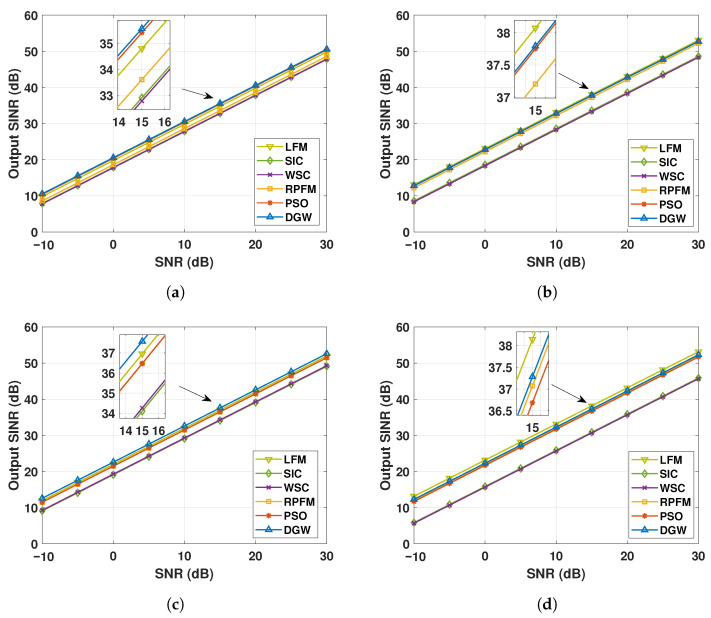
Comparison of output–input SNR for different algorithms under target/scene mismatches. (**a**) Output–input SNR performance under steering-vector mismatch. (**b**) Output–input SNR performance under wavefront distortion. (**c**) Output–input SNR performance under local coherent scattering. (**d**) Output–input SNR performance under local incoherent scattering.

To comprehensively evaluate the robustness of the proposed method, it is necessary to distinguish between algorithm-level robustness and system-level performance robustness under target- and scene-induced perturbations. Since only DGW and PSO belong to intelligent optimization frameworks with explicit fitness functions and iterative search mechanisms, this subsection first examines their convergence robustness from an optimization perspective, before further analyzing the output SINR performance of all algorithms under both ideal conditions and four mismatches scenarios, including steering vector mismatch, wavefront distortion, local coherent scattering, and local incoherent scattering as depicted in Figure 7, Figure 8 and Figure 9.

Specifically, the convergence behavior and statistical stability of the fitness function are analyzed to reflect the intrinsic robustness of the optimization algorithms with respect to random initialization and stochastic search processes. This analysis provides insight into whether the optimization algorithm can consistently converge to high-quality solutions, which is a prerequisite for reliable beamforming performance in practical applications. Figure 7 compares the convergence robustness and statistical stability of the proposed DGW algorithm with PSO method through multiple independent runs (here is 20). As shown in Figure 7a, the DGW method converges faster and exhibits a smaller standard deviation throughout the 30 iterations process, indicating both rapid convergence and reduced sensitivity to random initialization. In contrast, the PSO method shows slower convergence and larger fluctuation bands, reflecting higher uncertainty during the search process. The statistical distribution of the final fitness values is further illustrated in Figure 7b. The DGW method presents a more compact box with shorter whiskers and fewer outliers, demonstrating stable convergence behavior and strong robustness across repeated runs, whereas the PSO method exhibits a wider dispersion and occasional inferior solutions. These results confirm that the DGW method achieves not only faster convergence but also superior optimization robustness at the algorithmic level. Then, the robustness of all FO schemes is further evaluated at the system level by examining their output SINR performance under ideal conditions and various spatial–spectral mismatch scenarios.

Under ideal operating conditions, the DGW algorithm achieves an output–input SNR nearly matching the theoretical optimum provided by the LFM benchmark, demonstrating near-optimal performance. In comparison, PSO and RPFM show moderate degradations of approximately 0.81 dB and 1.07 dB, respectively, while SIC and WSC suffer more significant losses around 8 dB (Figure 8). These results indicate that DGW delivers the highest signal quality, providing a robust and reliable foundation for high-precision passive microwave array operation on static platforms.

Under steering-vector mismatch conditions, with the target and interfering sources deviating by $2^{\circ }$ in angle and 0.2 km in range, Figure 9a shows that the DGW algorithm maintains the highest output–input SNR, approximately 0.15 dB above the second-best PSO method. Both DGW and PSO exceed the theoretical optimum by about 0.7 dB, whereas SIC, WSC, and RPFM fall below the optimum, with WSC showing the largest degradation near 2 dB. These results confirm that DGW possesses superior robustness to target- and scene-induced spatial–spectral mismatches, enhancing passive microwave array performance.

As illustrated in Figure 9b, under wavefront distortion conditions, DGW maintains the best performance, with an output–input SNR only 0.28 dB below the theoretical optimum and slightly higher than PSO. SIC and WSC exhibit the poorest performance, approximately 3.6 dB lower than the third-ranked RPFM. This demonstrates that DGW effectively compensates for wavefront distortions, mitigating the impact of atmospheric turbulence on static-platform observations.

Under local coherent scattering conditions (Figure 9c), DGW achieves the highest output–input SNR, about 0.62 dB above the theoretical optimum. PSO and RPFM show comparable performance, roughly 0.48 dB below the optimum, while WSC and SIC remain the lowest, approximately 2.86 dB below the benchmark. These results indicate that DGW can effectively handle multipath effects in complex terrain, maintaining superior observation accuracy and robustness.

In the presence of local incoherent scattering (Figure 9d), DGW sustains the best performance, only 0.86 dB below the theoretical optimum and slightly outperforming PSO. SIC and WSC perform similarly but are approximately 6.44 dB lower than DGW. This confirms that DGW effectively mitigates the adverse effects of distributed clutter, ensuring reliable target detection.

Therefore, Figure 9 demonstrates that the proposed DGW algorithm consistently maintains superior performance under all four representative target- and scene-induced spatial–spectral mismatch conditions, highlighting its robustness and enhanced controllability for passive microwave array components on static platforms, and providing a reliable foundation for high-precision component-level detection and beamforming.

## 4. Conclusions

Passive microwave array components on static platforms face inherent limitations in spatial resolution and interference suppression due to fixed geometry and single-frequency excitation. This work proposes a nonlinear frequency-offset design method for passive microwave FDA-MIMO arrays using a Dingo–Gray Wolf (DGW) hybrid intelligent optimizer with a multi-metric fitness function that jointly optimizes mainlobe focusing, sidelobe suppression, and frequency-offset smoothness. Simulation results demonstrate that the proposed approach achieves high-resolution two-dimensional energy focusing, enhanced interference nulling, and robust performance under realistic target- and scene-induced spatial–spectral mismatches. These findings confirm that the method effectively improves the controllability, robustness, and target-detection capability of passive microwave array components on static platforms, offering a practical solution for high-resolution and reliable sensing in complex electromagnetic environments.

## Figures and Tables

**Table 1:** Disparities of algorithms’ mainlobe width in two dimensions.

Parameters	SIC	WSC	RPFM	PSO	DGW
Range (km)	5.55	4.25	2.4	1.3	0.26
Angle (degree)	5.57	5.57	5.57	5.57	5.57

## Data Availability

The original contributions presented in the study are included in the article, further inquiries can be directed to the corresponding author.
